# Stoßwellen für Genossen. Urologie als Thema in den Zeitungen der DDR (1946–1990)

**DOI:** 10.1007/s00120-026-02801-y

**Published:** 2026-04-17

**Authors:** Florian G. Mildenberger

**Affiliations:** Universitätsklinikum Düsseldorf, Moorenstr. 5, 40225 Düsseldorf, Deutschland

**Keywords:** Urologie nach 1949, Ärzte im Sozialismus, Neues Deutschland, Neue Zeit, Berliner Zeitung, Moritz Mebel, Medizinische Zeitgeschichte, Urology after 1949, Physicians in the eastern bloc, Neues Deutschland, Neue Zeit, Berliner Zeitung, Moritz Mebel, Contemporary history of medicine

## Abstract

Die Presse in totalitären Staaten bietet nur einen eingeschränkten Überblick bezüglich der gesellschaftlichen und auch gesundheitlichen Realität. Doch die Auswertung der drei wichtigsten Zeitungen der DDR (*Neues Deutschland, Neue Zeit, Berliner Zeitung*) ermöglicht einen Einblick, wie Propaganda letztlich vergeblich versucht, die Probleme im Gesundheitswesen zu kaschieren und den scheinbaren Erfolg des Sozialismus herauszustellen.

## Abstract (en)

The press in totalitarian states offers only a limited overview of social and health realities. However, an analysis of the three most important newspapers in the German Democratic Republic (GDR; *Neues Deutschland*, *Neue Zeit*, *Berliner Zeitung*) provides insight into how propaganda ultimately attempts in vain to conceal problems in the healthcare system and highlight the apparent triumph of socialism.

## Forschungsstand und Forschungsfrage

**Abb. 1:**
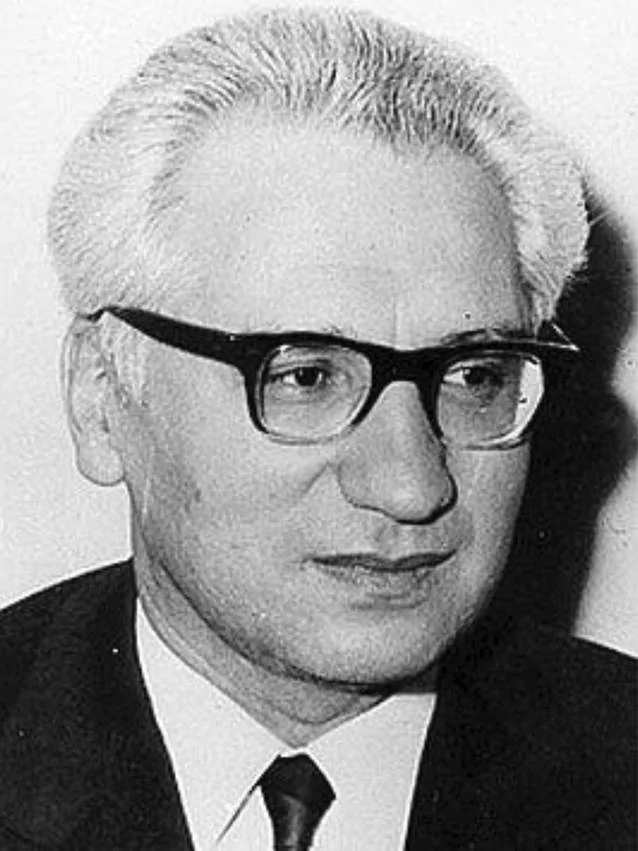
Moritz Mebel (1923–2021), Museum, Bibliothek und Archiv, Deutsche Gesellschaft für Urologie e. V. (Repro Moll-Keyn, mit freundl. Genehmigung)

Historische Forschung und zeitgenössische Wahrnehmung klaffen häufig auseinander. Denn gemeinhin entstammen die Historiker einem anderen Milieu als die Personen, die die Geschichte selbst erlebten. Für DDR-Bürger war beispielsweise klar, dass der bedeutendste Urologe ihres Landes Moritz Mebel (1923–2021) sein musste, da er ab 1970 Kandidat und seit 1986 Vollmitglied im Zentralkomitee der Sozialistischen Einheitspartei Deutschlands (SED) gewesen war. In den Publikationen der DGU nach 1990 spielte er jedoch nur eine marginale Rolle ([1, 2]; Abb. 1).

**Abb. 2:**
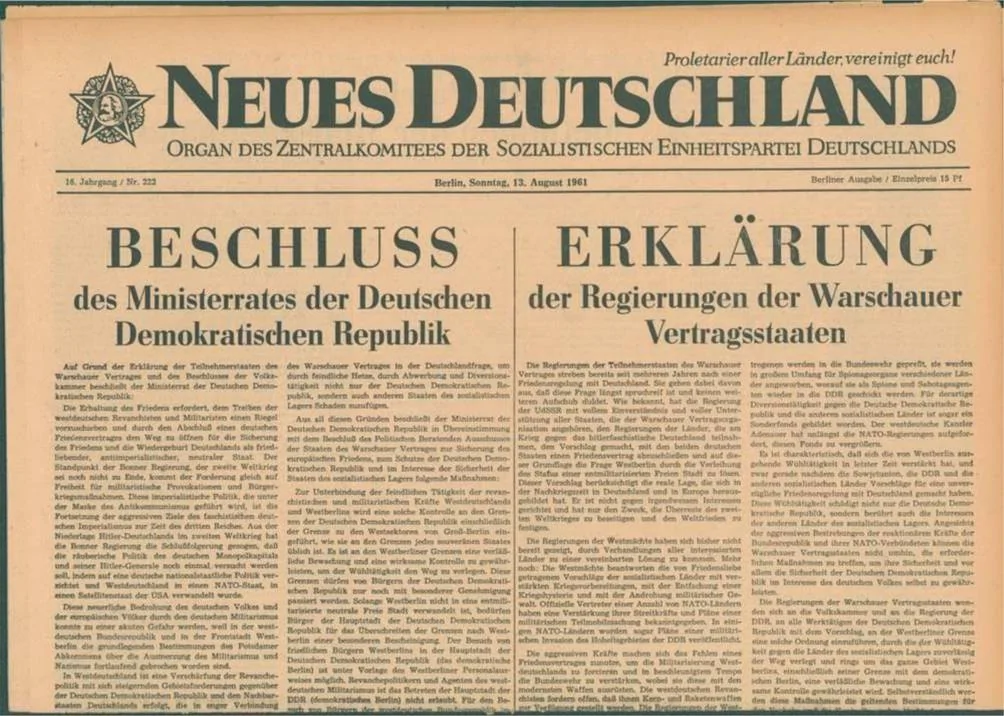
Cover des *Neuen Deutschlands*, entnommen aus DDR-Zeitungsportal der Staatsbibliothek zu Berlin

Die Historiographie konzentriert sich auf Kliniken, Entwicklungen in Forschung und Lehre, sozialhistorische Zusammenhänge und Biographien während die Darstellung in der Presse sich in gefeierten Erfolgen, „großen Ärzten“ und ideologischen Bezugspunkten erschöpft. Die Aufbereitung von Presseberichten für die zeithistorische Forschung war über Jahrzehnte problematisch, da die Digitalisierung von Tageszeitungen weder seitens der Deutschen Forschungsgemeinschaft (DFG) priorisiert wurde noch seitens der Historiker Wertschätzung erfuhr. Hinzu trat das Problem der Verwertungsrechte. Erst nachdem diese Fragen geklärt waren, erfolgte unter Federführung der Staatsbibliothek zu Berlin ab 2010 die Digitalisierung der bedeutendsten Tageszeitungen der DDR. Das *Neue Deutschland* (ND) fungierte als Zentralorgan der SED mit einer Auflage jenseits der Millionengrenze, die *Berliner Zeitung* (BZ) unterstand der lokalen Parteileitung und galt als etwas progressiver und verfügte immerhin über eine Auflage von 345.000 Stück. Die war sowohl Tageszeitung als auch Parteiorgan der Blockpartei CDU. Die Auflage schwankte im Laufe der Jahrzehnte zwischen 80.000 und 180.000. Man darf in der Rückschau nicht Parteizeitungen aus DDR und Bundesrepublik vergleichen. „*Vorwärts*“ oder „*Bayernkurier*“ konnten niemals Relevanz jenseits der Parteimitglieder erzeugen, während die Organe von SED oder CDU-Ost breite Leserschaften erreichten und insbesondere mit ihrem Wissenschaftsteil punkteten (Abb. 2).

**Abb. 3:**
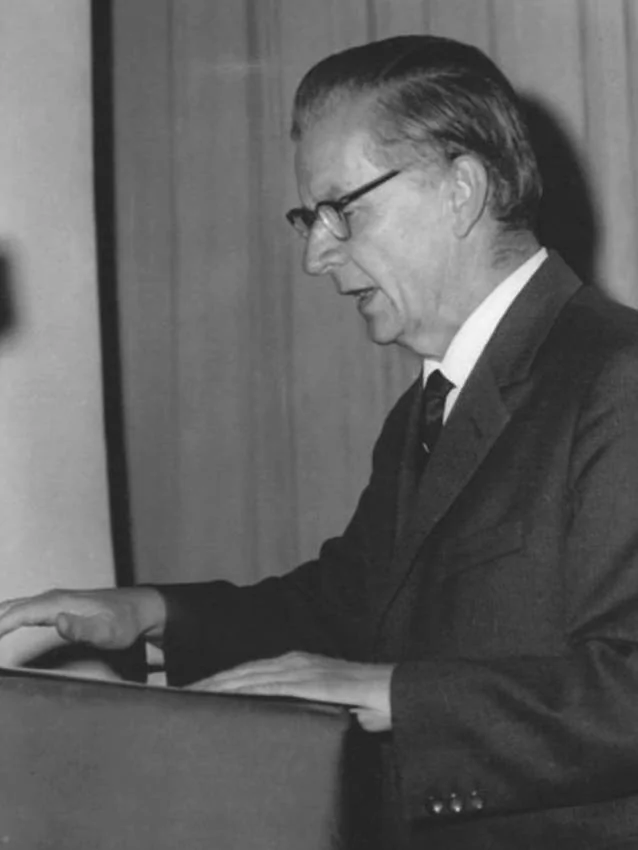
Emil Hienzsch (1914–1988) Deutsche Gesellschaft für Urologie e. V. (Repro Moll-Keyn, mit freundl. Genehmigung)

Das Ziel der Staatsbibliothek Berlin bei der Digitalisierung bestand darin, der Forschung einen „kritischen Umgang“ mit der DDR-Presse zu ermöglichen [3]. Insgesamt wurden etwa 400.000 Seiten mit mehr als 4 Mio. Artikeln erfasst und mit der Technologie des Frauenhofer Instituts für intelligente Analysen- und Informationssysteme (IADIS) verarbeitet. Die Fehlerquote der Scanner war (und ist) recht hoch, wie sich im Laufe des Forschungsvorhabens zeigte. So wird „Urologie“ bisweilen mit „Neurologie“ verwechselt. Gibt man nur „Urologie“ (mit Anführungszeichen) in das Suchportal ein, ergeben sich 440 Treffer für die Zeit von 1946 bis 1994 [4]. Nutzt man die Namen derjenigen Ärzte, die Fachhistoriker in ihren Aufsätzen als zentral einordneten, wird deren geringe Relevanz in der Presse der DDR schnell deutlich: der als zentral verortete Arzt Emil Hienzsch (1914–1988) wurde überhaupt nicht wahrgenommen, sein Berliner Kollege Ferdinand Hüdepohl (1902–1980) nur 3‑mal aufgeführt [5–7]. Auch die in der historischen Forschung untersuchten Lebenswege der Urologen Johannes Keller (1899–1970), Heinrich Götz (1908–2004) und Ernst Kirsch (1916–1990) waren für Zeitgenossen in der DDR offenbar weit weniger relevant, denn in den drei großen Tageszeitungen der DDR wurden sie kein einziges Mal erwähnt ([8–10]; Abb. 3).

Es mussten also Verdienste jenseits der medizinischen Kompetenz relevant gewesen sein, um in der (Partei)Presse der DDR als Urologe wahrgenommen zu werden.

## Urologie in der Presse bis zum Bau der Berliner Mauer

**Abb. 4:**
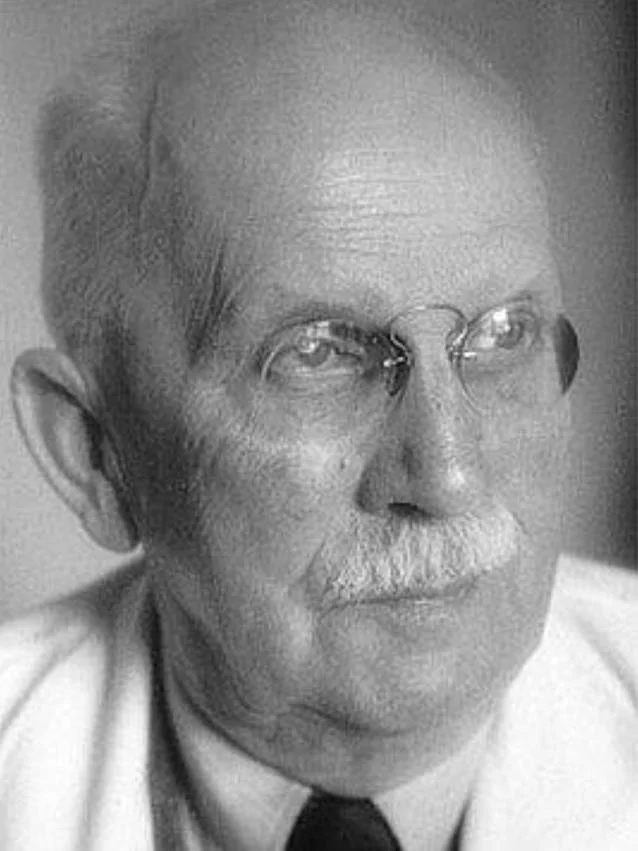
Walter Stoeckel (1871–1961), 1940er-Jahre Deutsche Gesellschaft für Urologie e. V. (Repro Moll-Keyn, mit freundl. Genehmigung)

Als sich in der zweiten Hälfte der 1940er-Jahre die „Sowjetische Besatzungszone Deutschlands“ (SBZ) allmählich in die selbständige „Deutsche Demokratische Republik“ (DDR) verwandelte, begann die SED als führende politische Kraft sukzessive sämtliche Aspekte und Berufsgruppen des öffentlichen und privaten Lebens zu durchdringen. Infolgedessen erließ die SED 1947 eigene „gesundheitspolitische Richtlinien“, die auf eine Synthese aus sowjetischem Gesundheitssystem und abgerissenen Reformplänen der Arbeiterpartien in der Zeit der Weimarer Republik abzielten, wobei die eugenische Komponente diskret ausgespart wurde [11]. Langfristiges Ziel war die Entfaltung dessen, was Historiker in den 1990er-Jahren eine „Fürsorgediktatur“ nennen sollten: staatlich bestallte Ärzte in Polikliniken und Ambulatorien würden die niedergelassene Ärzteschaft ablösen und die überlasteten und unterbesetzten Kliniken entlasten [12]. Die SED war an der Ausbeutung der Arbeitskraft interessiert, nicht grundsätzlich an der Gesundheit der Bürger, wie der Sozialmediziner Kurt Winter (1910–1987) recht offen zugab. So sei das Ziel der Gesundheitspolitik, „für jeden Bürger die Voraussetzungen zu schaffen, die heute mögliche Lebenserwartung voll und in gesellschaftlicher Aktivität ausschöpfen zu können. Mit anderen Worten: Die Medizin, die Gesellschaft und alle Bürger müssen sich gemeinsam bemühen, Lebensalter und Leistungsalter in Übereinstimmung zu bringen.“ [13]. Vorerst war die Ärzteschaft voll mit der Niederkämpfung von kriegsbedingten Mangelerkrankungen und Tuberkulose beschäftigt. Doch in den 1950er-Jahren machte sich der „brain drain“ durch Abwanderung in den Westen stark bemerkbar. Dies führte dazu, dass die Förderung von Spezialdisziplinen durch das knauserige Ministerium für Gesundheitswesen erheblich in den Hintergrund geriet. Stattdessen sollten die Angehörigen der „großen“ Fachgebiete sozusagen „Alleskönner“ sein. Dies drückte sich auch in der Darstellung in der Presse aus. So wurde der Gynäkologe Walter Stoeckel (1871–1961) gelobt, da er nicht nur Frauen behandelte, sondern auch eine Koryphäe auf dem Gebiet der „urologischen Gynäkologie“ sei [14]. Einheimische Erfinder, die tragbare Röntgengeräte für den Einsatz in gynäkologischen, pädiatrische und urologischen Praxen erdachten, wurden mit Lob überschüttet ([15, 16]; Abb. 4).

Wurden ärztliche Tagungen in der Presse erwähnt, so wurde betont, dass hier nicht nur Vertreter eines Faches – z. B. der Chirurgie – diskutierten, sondern auch andere Fachgebiete wie Urologie mit erfasst wurden [17]. Doch weil offizielle Propaganda und gesellschaftliche Realität immer weiter auseinander klafften, vollzog sich Ende der 1950er-Jahre ein Wandel. Nun wurden auch Tagungen von Urologen als solche vorgestellt [18, 19] und wenn Chirurgen urologische Themen diskutierten, fanden sich plötzlich Formulierungen wie „Das Gebiet der Urologie, das heute auch in der DDR immer mehr zum Spezialfach wird …“ [20]. Indirekt gab die Parteipresse damit zu, dass die Konzeptionen des Ministeriums von 1949, wonach „Alleskönner“ die Interessen der Patienten bedienen würden, gescheitert war – vermutlich weil die entsprechenden Ärzte längst im Westen praktizierten. Aber noch in Nachrufen auf Stoeckel, dessen NS-Vergangenheit gänzlich tabuisiert wurde, fand sich der Hinweis auf seine führende Rolle als „gynäkologischer Urologe“ [21, 22]. Der Bau der Berliner Mauer beendete den „brain drain“, erhöhte jedoch auch den Druck auf die Parteiführung, den Versprechungen auf eine bessere Zukunft rasch Taten folgen lassen zu müssen. Davon profitierten bislang vernachlässigte Berufsgruppen wie die Ärzteschaft, deren Ausbildungs- und Arbeitsbedingungen massiv verbessert wurden [23].

## Der Aufstieg der Urologen

**Abb. 5:**
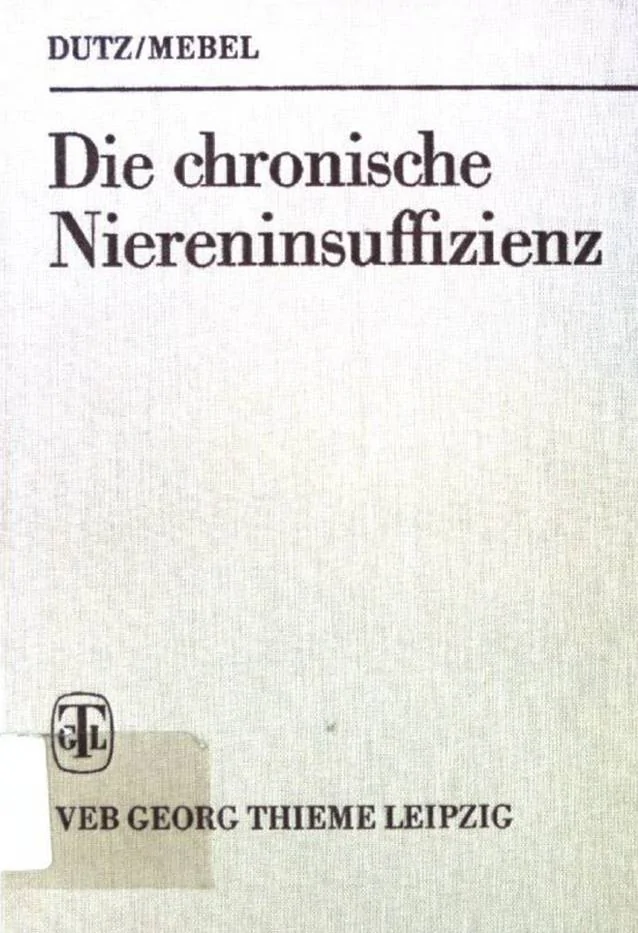
Dutz H, Mebel M (1977) Die chronische Niereninsuffizienz VEB Georg Thieme Leipzig. (Repro Moll Keyn, mit freundl. Genehmigung)

Um nicht von den Chirurgen vereinnahmt zu werden, gründete sich nach einer vorbereitenden Arbeitstagung im Dezember 1961 im Frühjahr 1962 die „Arbeitsgemeinschaft der Urologen in der DDR“, aus der 1968 die „Gesellschaft für Urologie in der DDR“ hervorgehen sollte [24]. Die Urologen fügten sich in die politischen Vorgaben auf ihre Weise ein. So zog Emil Hienzsch in Jena gezielt urologischen Nachwuchskräfte heran, die sich daraufhin rund um die Universitätsstadt als Fachärzte niederließen bzw. in staatliche Praxen eintraten [24]. Am grundsätzlichen Misstrauen der SED gegenüber den (Fach)ärzten endete dies nichts [25]. Ob bei den Prüfungen unterschwellig auch die politische Haltung überprüft wurde, ist umstritten [26]. Einen Versorgungsgrad, wie ihn die WHO als Standard vorgab, erreichte die Urologie in der DDR erst 1974 [27]. In der Parteipresse jedoch vollzog sich ein rapider Wandel in der Darstellung des Fachgebiets und seiner Vertreter. Waren sie bislang bestenfalls als Hilfskräfte von Chirurgen präsentiert worden, so waren sie nun eine angesehene Gruppe an Fachärzten geworden. Ihre Tagungen wurden als akademische Veranstaltungen eines festgelegten Fachbereichs dargestellt [28–30] und die Leistungen der Urologen als so bedeutend herausgestellt, dass auch sowjetische Kollegen gerne zu Besuch kämen [31]. Im Fall einer Tagung wurde extra vermerkt, dass Gäste aus „Westdeutschland“ zur Weiterbildung in die DDR gekommen seien [32]. 1967 avancierte dann ein Urologe erstmals zur Hauptperson im Rahmen einer Reportage des *Neuen Deutschlands* über die Verhältnisse in den Kliniken. Rein zufällig angetroffene Patienten betonten „Ich bin von Prof. Mebel ganz begeistert“ und sogar die Reinigungskräfte zeigten sich von der Freundlichkeit des Urologen Moritz Mebel äußerst angetan [33]. Er sollte alsbald eine zentrale Rolle in der Außenwahrnehmung seines Faches spielen. Denn in Kenntnis der ungeliebten Tatsache, dass die sozialistische DDR der Bundesrepublik auf allen ökonomischen Ebenen unterlegen war und sie niemals einholen würde, kaprizierten sich die Strategen des Planungsapparats auf einzelne Aspekte aus Wissenschaft, Sport oder Gesellschaftspolitik, auf denen die DDR dem „Klassenfeind“ mindestens ebenbürtig war. Hier spielte die Nierentransplantation eine zentrale Rolle. Sie wurde bereits 1968 als zukunftsweisendes Arbeitsgebiet der Urologie präsentiert [34, 35]. Die Universitätsklinik Halle hatte seit 1959 auf diesem Gebiet wegweisende Forschungen erbracht, eine künstliche Niere entwickelt und die Grundlagen für den Aufbau von Dialysezentren gelegt [36]. Den Ruhm hierfür erntete jedoch nicht „Team Halle“ sondern die Urologische Klinik am Krankenhaus im Friedrichshain in Ost-Berlin. So konnte man der *Berliner Zeitung* entnehmen, dass nicht Halle, sondern Berlin-Friedrichshain der Geburtsort der Nierentransplantation in der DDR gewesen sei, wodurch das Land „zu den führenden Ländern“ auf diesem Gebiet weltweit avanciert sei [37]. Sein Leiter Moritz Mebel wurde als sozialistisches Vorbild präsentiert [38], seine politische Verortung als „Kandidat der Nationalen Front“ hervorgehoben [39] und seine zentrale Rolle als Wegbereiter einer modernen sozialistischen Urologie benannt [40–42]. Tatsächlich zeichnete Mebel verantwortlich für die entsprechenden Lehrbücher und hielt hierüber auch einen Vortrag vor der Akademie der Wissenschaften ([43–46]; Abb. 5, 6 und 7).

**Abb. 6:**
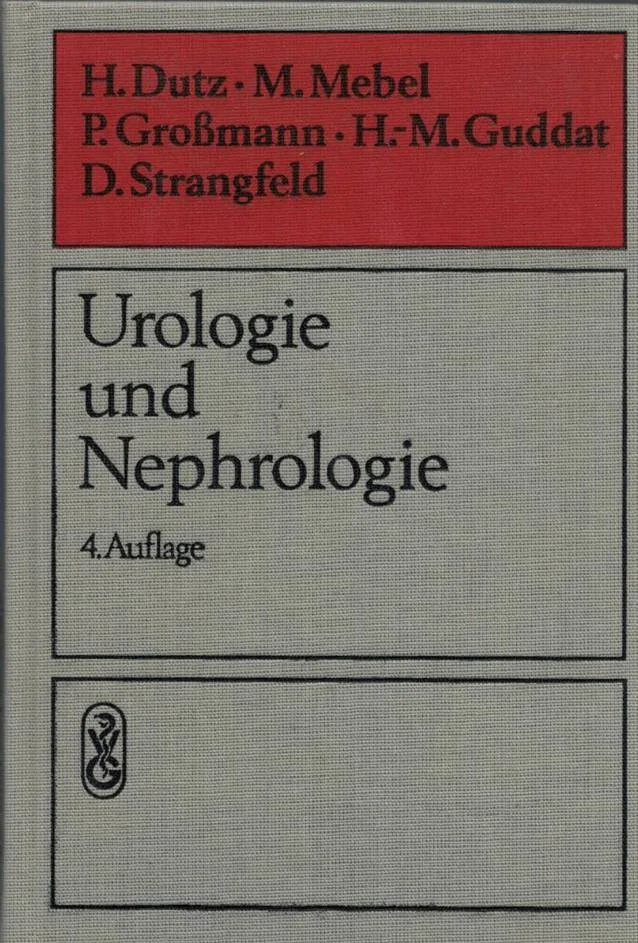
Dutz H, Mebel M u. a. (1978) Urologie und Nephrologie. Ein Leitfaden für Studenten. (VEB Verlag Volk und Gesundheit, Ost-Berlin, Repro Mildenberger, mit freundl. Genehmigung)

**Abb. 7:**
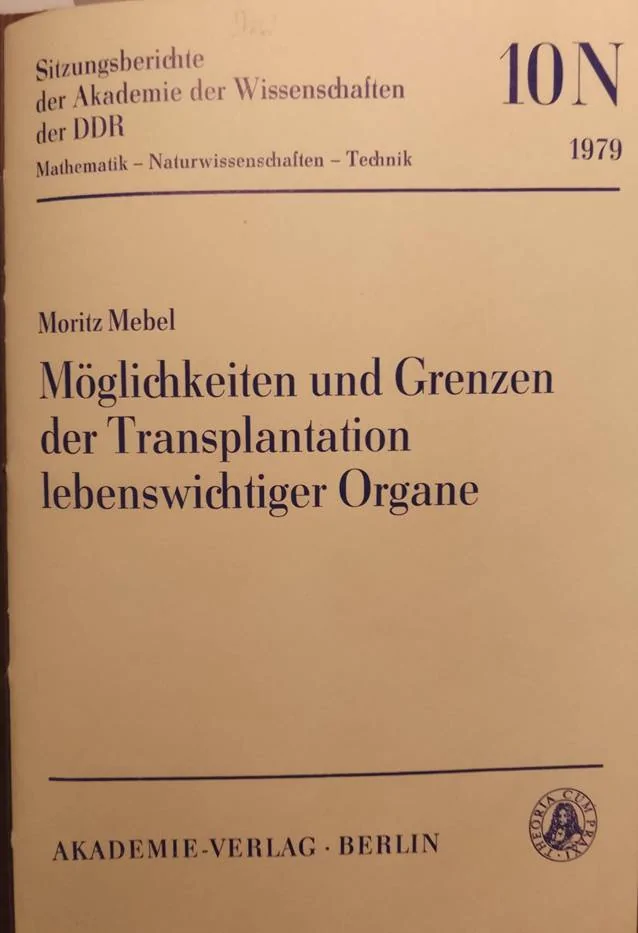
Mebel M (1977) Möglichkeiten und Grenzen der Transplantation lebenswichtiger Organe. Akademie der Wissenschaften der DDR, Ost-Berlin

Auch präsentierte sich der vormalige Exilant Mebel als politisches Vorbild und unverbrüchlicher Freund der Sowjetunion [47, 48]. Ehrungen folgten und in den 1980er-Jahren tourte Mebel im Parteiauftrag quer durch die Welt um für Nierentransplantationen, DDR-Medizintechnik und den Weltfrieden zu werben [49–53]. Schließlich wurde er noch zum Begründer der Nierensteinzertrümmerung erhoben [54, 55]. Zeit seines Lebens betonte Mebel den hohen Standard der DDR-Urologie, die Versorgungssicherheit für Patienten und die prinzipielle Überlegenheit des Sozialismus gegenüber dem Westen [56]. Doch die Realität sah etwas anders aus und dies ließ sich auch in der Parteipresse zwischen den Zeilen erkennen.

## Anspruch und Wirklichkeit

**Abb. 8:**
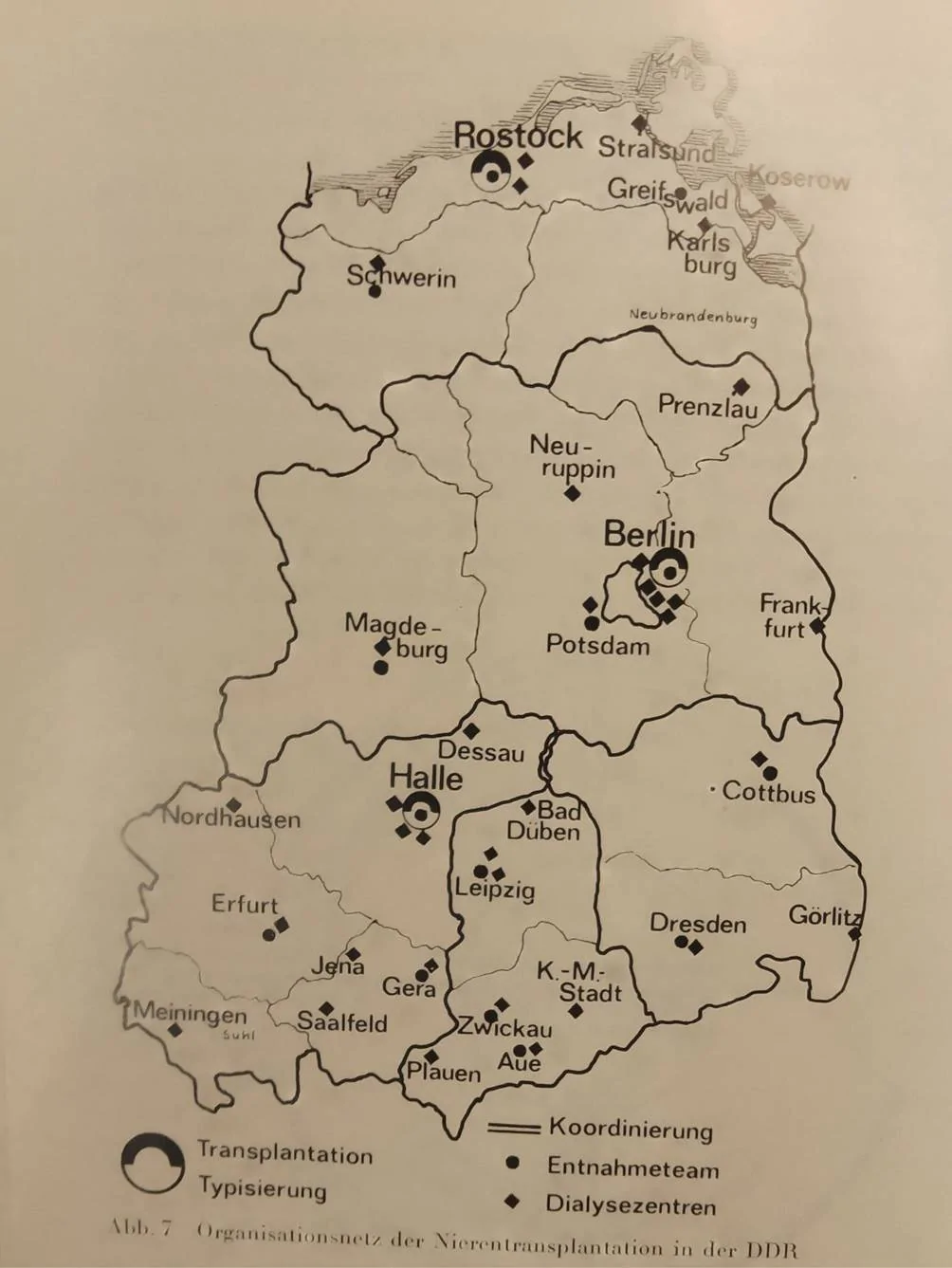
Dialysezentren und Nieren-Entnahmeteams in der DDR (entnommen aus Dutz H, Mebel M u. a. [1978] Urologie und Nephrologie. VEB Verlag Volk und Gesundheit, Ost-Berlin, S 35)

Vordergründig gestaltete sich die Situation für urologische Ärzte und Patienten in der DDR der 1970er-Jahre höchst positiv. 115 ambulatorische Einrichtungen versorgten die Kranken und Urologie war fester Bestandteil der Ärztefortbildungen [57]. Doch den Beamten im Ministerium war gleichwohl die massive „fachärztliche Unterversorgung“ bekannt [58]. In weiten Teilen des Landes gab es überhaupt keinen Urologen. Gleichwohl behauptete die Parteipresse die Existenz einer hervorragenden Versorgungslage. Um diese zu untermauern, verging kaum ein Monat, in dem nicht auf die Eröffnung einer neuen Poliklinik verwiesen wurde. So wurden die Eröffnungen der Versorgungseinrichtungen inklusive urologischer Fachärzte in Rostock, Hoyerswerda, Ludwigsfelde, Wittenberg und Elsterwerda gebührend gefeiert ([59–63]; Abb. 8).

**Abb. 9:**
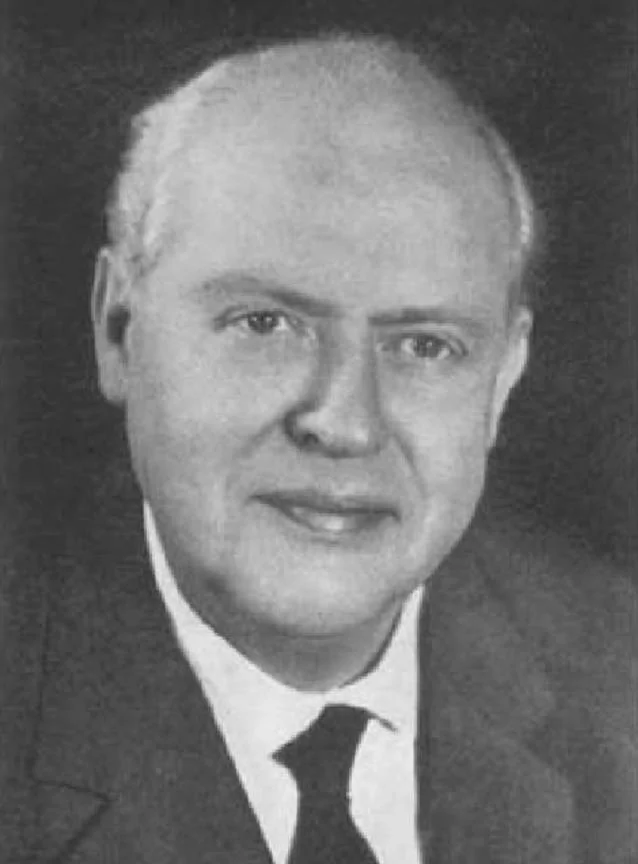
Gerhard Heise (1911–1982), Deutsche Gesellschaft für Urologie e. V. (Repro Moll-Keyn, mit freundl. Genehmigung)

Dadurch aber gaben die Parteijournalisten indirekt zu, dass die angeblich perfekte Versorgungssituation im Sozialismus erst noch geschaffen werden musste und keineswegs bereits existierte. Gespräche mit „verdienten Ärzten“ ergaben, dass die urologische Sprechstunde stets überfüllt war, wodurch sich zwangsläufig die Frage stellte, warum die Patienten nur einen einzigen Anlaufpunkt hatten [64]. Sogar der Lehrstuhlinhaber für Urologie an der Medizinischen Akademie Magdeburg, Gerhard Heise (1911–1984) räumte in einem Interview ein, dass es in vielen Bezirken der DDR keinen Arzt gab, an den sich Patienten mit urologischen Problemen wenden konnten ([65]; Abb. 9).

**Abb. 10:**
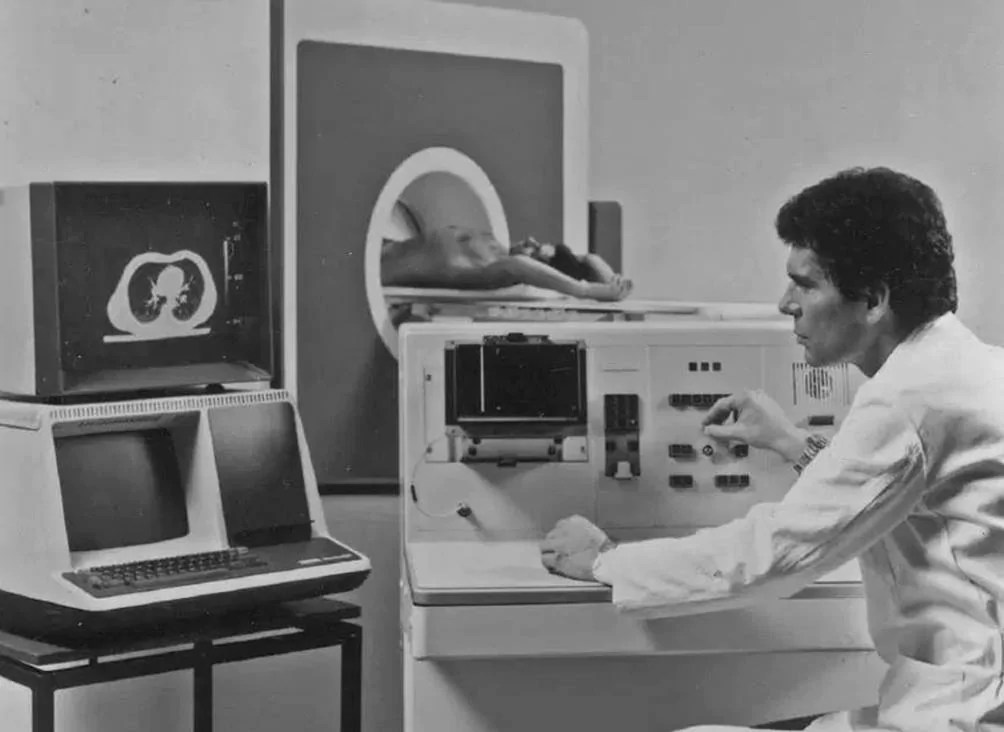
Siemens Somatom 1977 Deutsche Gesellschaft für Urologie e. V. (Repro Moll-Keyn, mit freundl. Genehmigung)

Selbst in der Hauptstadt Ost-Berlin geriet die simple Eröffnung einer urologischen Arztpraxis zum Medienereignis [66, 67]. Auch eine Meldung wie die, dass das eigentlich nur zur banalen Gesundheitsaufklärung geplante „Haus der Gesundheit“ am Berliner Alexanderplatz vorrangig als Versorgungszentrum fungierte, ließ tief blicken [68]. Dass die Einrichtung eines Desinfektionszentrums 1978 eine Schlagzeile wert war, wies ebenfalls auf grundlegende Missstände hin [69]. Renovierungen an der Charité in Ost-Berlin wurden als bedeutend für die Urologie gefeiert – so als ob ein moderner Operationssaal nicht eine Selbstverständlichkeit wäre [70, 71]. Um über die triste Situation hinwegzutäuschen, beschworen die sozialistischen Edelfedern das exzellente Arbeitsverhältnis zwischen Schwestern und Ärzten und die optimalen Weiterbildungsmöglichkeiten für erstere [72–74]. Trotzig wurde die internationale Vernetzung und die Führungsrolle der DDR auf dem Gebiet der Nierentransplantation betont [75–77]. Doch spätestens zu Beginn der 1980er-Jahre verlagerte sich die Diskussion um die Möglichkeiten der Kliniken und Ärzte von den Parteizeitungen zu einem vormodernen Medium: der Eingabe einfacher Bürger an die Spitzenvertreter des Staates. Das ZK der SED wurde jedes Jahr mit mehr als 750.000 Beschwerden über die Verhältnisse im real existierenden Sozialismus konfrontiert. Das Ministerium für Gesundheitswesen allein erhielt 1980–1987 jedes Jahr 30.000 Briefe über Missstände und Versorgungsmängel [78]. Die technologische Rückständigkeit gegenüber dem Westen zeigte sich immer deutlicher, wobei hier die Wissenschaftsprogramme im Westfernsehen eine verheerende Rolle spielten. Vergeblich versuchte die Gesellschaft für Urologie in der DDR durch eine Kooperation mit der Republik Österreich an dringend benötigte Technologie zu gelangen [79]. Die ersten Computertomographen erreichten die Kliniken 1987/88 ([80]; Abb. 10).

**Abb. 11:**
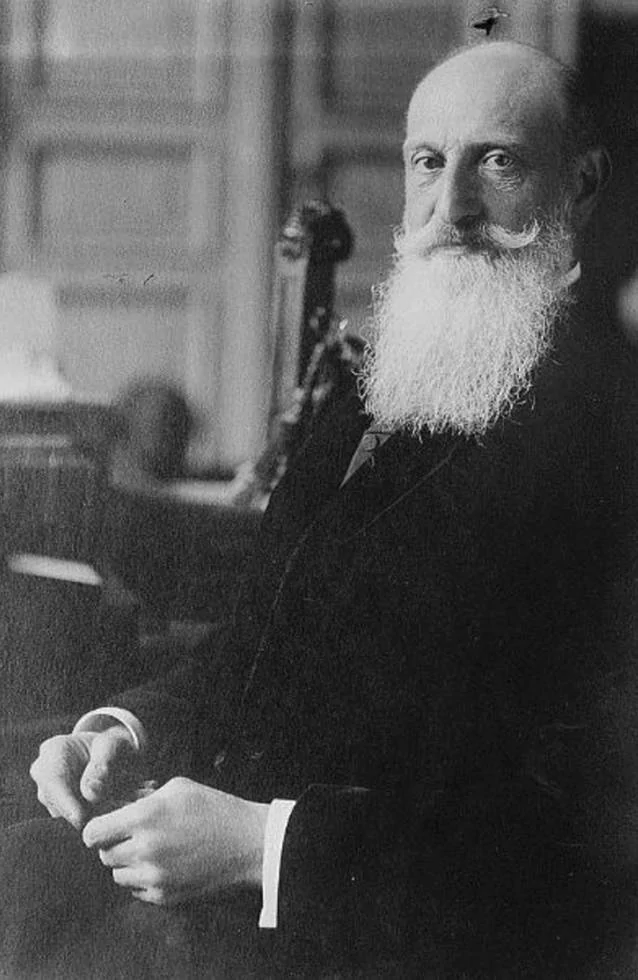
James Israel (1848–1926) Deutsche Gesellschaft für Urologie e. V. (Repro Moll-Keyn, mit freundl. Genehmigung)

Da die Aufrüstung aller ärztlichen Fachdisziplinen mit modernsten Geräten als unfinanzierbar angesehen wurde, kaprizierte sich das Ministerium für Gesundheitswesen auf die Idee, verschiedene Fachrichtungen zu einer verstärkten Kooperation zu zwingen, was in der Presse als großer Wurf präsentiert wurde [81, 82]. Im Grunde kehrte man damit medizinalpolitisch in die 1950er-Jahre zurück. Um dem Ärztemangel zu begegnen, wurden sogar Pensionisten aus den Blockparteien verpflichtet, gemeinsam mit Fachärzten für Urologie Patienten in speziellen Sprechstunden zu versorgen [83]. Denn die Zahl an Patienten nahm zu, u. a. weil in der Parteipresse dafür geworben wurde, bei jedem Unwohlsein sogleich einen Facharzt aufzusuchen [84]. Das System begann sich selbst zu strangulieren. Um die politisch beworbenen, aber höchst kostenintensiven Nierentransplantationen zu begrenzen, wurde nun die „Nierenkonservierung“ empfohlen [85]. Selbst die Jubelmeldungen über neue Einrichtungen gerieten zur Farce, z. B. als das Bezirkskrankenhaus Görlitz 1980 sein 25-jähriges Bestehen feierte und der Journalist dabei durchblicken ließ, dass es nicht nur das beste urologische Versorgungszentrum in Ostsachsen darstellte, sondern v. a. das einzige [86]. Selbst vor den Toren der Hauptstadt Ost-Berlin in Treptow wurde die Niederlassung eines Urologen als herausragender Erfolg des Gesundheitswesens beworben [87]. Parallel aber überschütteten die Parteiblätter die Leser mit Mitteilungen über die grandiosen Erfolge der sozialistischen Urologen auf nationalen und internationalen Tagungen [88–90]. In den 1980er-Jahren begann im gesamten Ostblock auch die Erinnerung an das „Erbe der Arbeiterbewegung“ in der Zeit vor Hitler und Stalin Gestalt anzunehmen. Diese Entwicklung ging an den Urologen der DDR nahezu spurlos vorüber. Nur 1982 wurde an den „Wegbereiter der Urologie“ James Israel (1848–1926) kurz erinnert ([91]; Abb. 11 und 12).

**Abb. 12:**
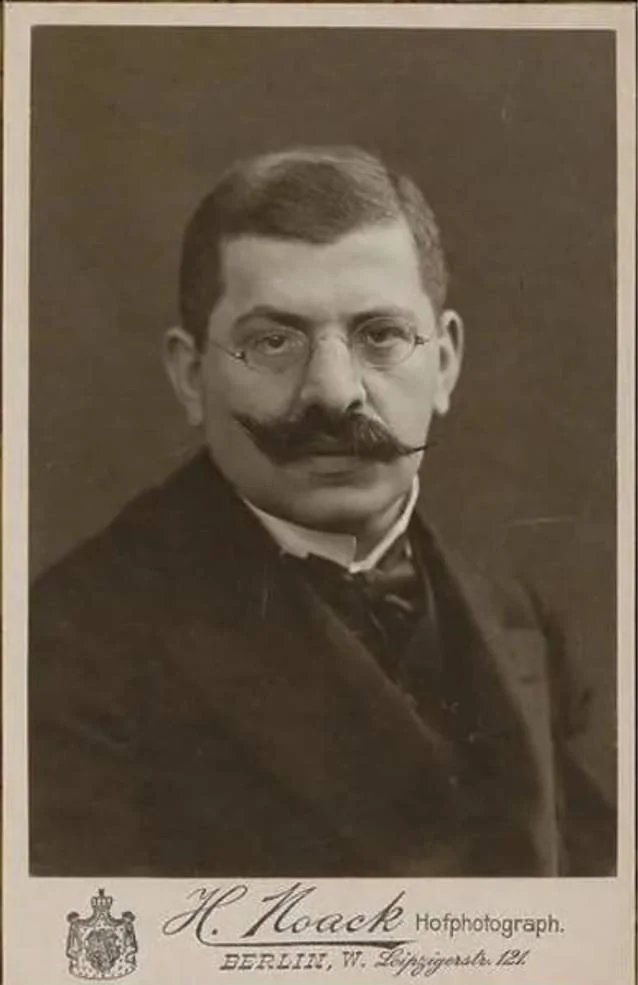
Magnus Hirschfeld (1868–1935), um 1900, Carte de visite, Hof-Photograph der I M der Königin von Preußen, Berlin Leipziger Straße, vormals Unter den Linden 34 3. Etg. Deutsche Gesellschaft für Urologie e. V. (Repro Moll-Keyn, mit freundl. Genehmigung)

Ähnlich verhielt sich die Situation mit der Weimarer Sexualforschung. Obwohl sich die DDR als Erbe der deutschen Arbeiterbewegung und ihrer Verbündeten aus der Zeit vor dem Nationalsozialismus verstand, wurden die engen Bande zwischen der politischen Linken und bedeutenden Gelehrten wie Magnus Hirschfeld (1868–1935) in den 1980er-Jahren höchst zögerlich thematisiert [92, 93].

## Ende ohne Anfang

Erst Mitte November 1989, als die Berliner Mauer schon gefallen war, rang sich ein Journalist in der *Neuen Zeit* dazu durch, das Vorzeigeprodukt der DDR-Urologie, die Nierentransplantationen als Farce zu benennen. Patienten mussten meist 26 Monate auf ein Spenderorgan warten, so dass die hohen Erfolgszahlen v. a. darauf beruhten, dass die schwierigen Fälle die Operation gar nicht mehr erlebten [94]. Der vormalige Klinikdirektor Moritz Mebel musste sich alsbald kritische Nachfragen gefallen lassen, woher er seine „lebensfrischen“ Nieren bezog. Offenbar ließ er transportunfähige potentielle Spender in seine Klinik verfrachten, um sie dann nach dem erwarteten Ableben ausweiden zu können [95]. Erster Profiteur der noch 1989 angeschafften Westtechnik für die urologische Station der Charité wurde ironischerweise Erich Honecker (1912–1994), der dort von Peter Althaus betreut wurde [96]. Das Ministerium für Staatssicherheit büßte seine Spezialkliniken ein, wovon auch Urologen profitierten [97]. Die beschworene Einheit von Ärzten und Krankenschwestern entpuppte sich als Propaganda und entlud sich alsbald in Streiks des Pflegepersonals, worüber die Journalisten in den vormaligen Parteiblättern berichteten ohne auf ihre früheren Einlassungen einzugehen [98]. Für den früheren Star der DDR-Urologie Moritz Mebel bedeutete die Wende das endgültige Ende seiner Karriere und Medienpräsenz. Da er als Angehöriger des ZK der SED in der Sitzung vom 18. Oktober 1989 im Kontext der Ablösung Honeckers durch Egon Krenz zarte Worte der Kritik verlauten hatte lassen, entdeckte ihn die „Berliner Zeitung“ als Vertreter eines „wahren“ Sozialismus und bot ihm in den folgenden Jahren ein Forum [99, 100]. In einem Interviewband gewährte Mebel 1999 Einblick in sein Leben und ließ die Leser wissen, dass ihn an der Politik der DDR lediglich Ausbürgerung von Genossen und das „Freikaufen“ von Oppositionellen durch die Bundesrepublik gestört hätten [101]. Nach seinem Tod 2021 erhielt er im *Neuen Deutschland* einen bombastischen Nachruf in dem nahezu ausschließlich auf Mebels Leben vor 1949 und insbesondere als Kämpfer auf der Seiten der Roten Armee im Zweiten Weltkrieg eingegangen wurde [102]. So als ob es die DDR und die Urologie der DDR nie gegeben habe. Es wurde, wie schon Zeitgenossen spöttisch bemerkten, „gekonnt vermebelt“.
